# Diet Quality, Supplementation, and Professional Support as Markers of Injury Load in Polish Football Players: A Cross-Sectional Study

**DOI:** 10.3390/nu18060887

**Published:** 2026-03-11

**Authors:** Aureliusz Kosendiak, Dawid Konieczko, Elżbieta Biernat

**Affiliations:** 1 Institute of Health Studies, University of Lower Silesia, 53-611 Wroclaw, Poland; aureliusz.kosendiak@dsw.edu.pl; 2 Medical Faculty, Wroclaw Medical University, 50-345 Wroclaw, Poland; 3 Tourism Economy Research Unit, Institute of International Economic Policy, Collegium of World Economy, SGH Warsaw School of Economics, 02-554 Warsaw, Poland; ebiern1@sgh.waw.pl

**Keywords:** American football players, diet, supplementation, dietary and physiotherapy services, injuries, pain, Poland

## Abstract

**Background/Objectives**: American football (AF) is a contact sport with a high injury risk, where an integrated model of interdisciplinary care may support recovery. The aim of this study was to assess the relationship between diet quality, supplementation, dietician’s and physiotherapist’s support, injury rates and musculoskeletal pain among AF players in Poland. **Methods**: The study involved 53 male players from the FA Panthers Wrocław team and was conducted using the KomPAN questionnaire and an original survey on supplementation, injuries and pain intensity. **Results**: The results showed that, although players using supplements had a significantly greater proportion of healthy foods in their overall diet (*p* = 0.02), they did not report fewer injuries in the last 12 months and 7 days than those who did not use supplements (*p* = 0.87; *p* = 0.58, respectively). However, a positive correlation (*p* ≤ 0.001, r = 0.53) was found between the healthy diet index and the number of injuries. Those who used the services of a dietitian and physiotherapist showed a higher quality of diet (*p* = 0.02; *p* = 0.02, respectively) and reported higher total pain intensity (*p* = 0.009; *p* = 0.03), while those who used only the services of a physiotherapist reported higher average pain intensity (*p* ≤ 0.001). **Conclusions**: These results suggest that supplementation and professional support in this group are primarily due to exposure after injuries caused by severe pain, rather than serving a preventive function.

## 1. Introduction

American football (AF) is a high-intensity intermittent collision sport, combining short bursts of maximal exertion with intense physical contact and brief rest intervals. It requires exceptional strength, power, and endurance. Frequent collisions, rapid changes in direction, and physical overload make players particularly susceptible to musculoskeletal injuries and overuse conditions [1]. According to data from the National Collegiate Athletic Association [2], as many as 70–80% of players experience injuries during the season, although this figure may be lower depending on the specific context. An integrated interdisciplinary care model—encompassing collaboration among coaches, dietitians, physiotherapists, and physicians—can effectively mitigate injuries in many disciplines and support player performance and recovery [3,4].

Although research regarding comprehensive support in AF is lacking, analyses of contact sports demonstrate that insufficient intake of energy, protein, and key micro- and macronutrients prolongs recovery, exacerbates inflammation, and increases the risk of injury [5,6]. Inversely, adequate nutrition and supplementation help limit injuries, inflammation, and post-exercise pain [7,8]. Additionally, supplementation with omega-3 fatty acids eases delayed onset muscle soreness [9]. For these reasons, among others, athletes in contact sports (including AF [10]) often utilize ergogenic, regenerative, and protective agents.

A key factor influencing athletes’ diet and supplementation, and consequently the incidence of injuries and musculoskeletal overload, is nutritional awareness [11]. A high level of awareness promotes better dietary choices, rational supplementation, and injury prevention, while a low level leads to nutritional errors and slower recovery [5,6]. Abbey et al. [12] demonstrate that AF players not only often possess fragmentary knowledge in this area but also rarely utilize professional support. Similar observations are presented by Sobek et al. [13]—according to their research, only 12% of players regularly seek advice from dietitians. As a result, athletes’ diets often prove to be unbalanced, and the use of supplementation is inadequate, which may lead to reduced performance and decelerated recovery processes [12,14].

Physiotherapists occupy an important place in preparation for exertion and the health protection of AF players. They are responsible for injury monitoring, rehabilitation, and clearing athletes for return to play following injury [15]. Their collaboration with coaches and dietitians significantly accelerates the return to full physical fitness [16,17], although this topic is not sufficiently understood [16]. It should be mentioned that, in addition to conducting functional therapy, they also participate in the process of education on regeneration, training loads and energy requirements [18]. For this reason, contact with physiotherapists may indirectly influence nutritional behavior through increased awareness of metabolic needs and more frequent contact with an interdisciplinary team. The use of physiotherapy can therefore be considered an indicator of medical care related to sports injuries, although this topic has not yet been sufficiently researched. Generally, the current literature lacks studies covering the full range of factors influencing the recovery of AF players and their athletic disposition.

Therefore, the aim of this study is to determine the relationship between dietary choices, nutritional awareness, use of dietitian and physiotherapist services, injury incidence and musculoskeletal pain severity among AF players in Poland.

The following hypotheses were postulated:

**H1.**  *Supplement users are characterized by higher diet quality and greater nutritional awareness;*

**H2.**  *Supplement users differ from non-users in terms of the number of injured sites and the number of sites with a specific pain intensity;*

**H3.**  *A higher level of healthy nutritional behaviors (more regular meals, better diet, and less frequent snacking during the day) correlates with a lower number of injuries and lower pain intensity;*

**H4.**  *Utilizing the services of a physiotherapist after injury and a dietitian is associated with higher diet quality, lower pain intensity, and a higher number of injured sites.*

The results may fill the existing research gap by providing data on the importance of an integrated, interdisciplinary approach to injury prevention and recovery in this demanding discipline.

The existing studies have focused mainly on individual factors related to sports injuries, analyzing diet, supplementation, or medical support separately; also there is a lack of studies that cover the full range of factors affecting the recovery of FA players and their athletic performance. In addition, the available data on the physically active population in Poland are limited.

## 2. Materials and Methods

### 2.1. Population, Iclusion and Exclusion Criteria

**Figure 1:**
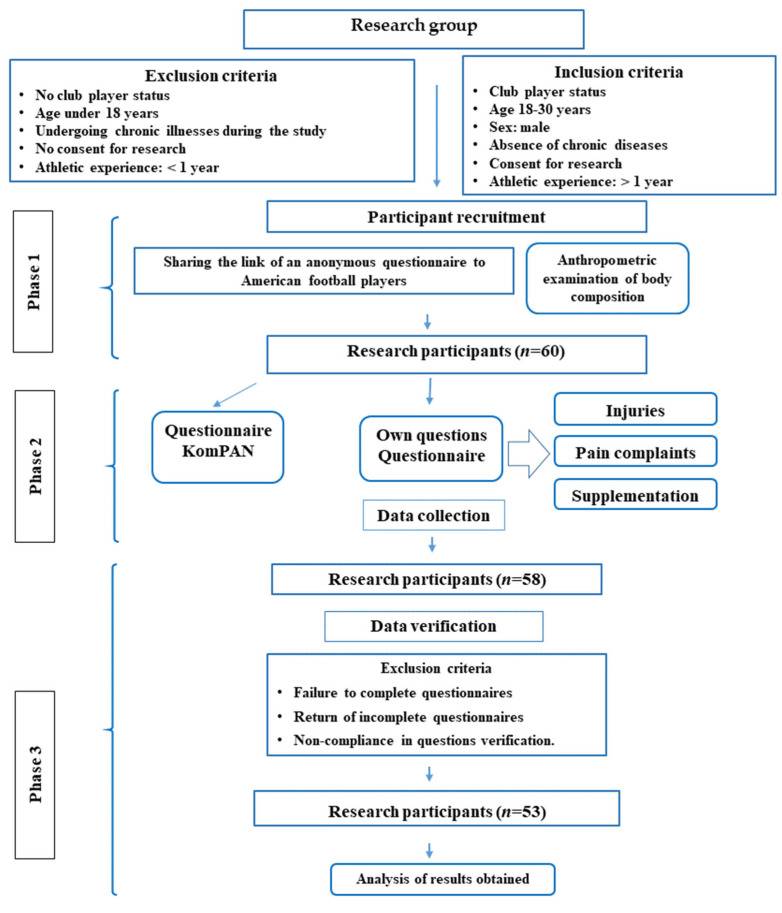
Research process among American football players.

The study was conducted in March 2021. The participants were male active players of the professional AF Panthers Wrocław team (*n* = 53). The study was conducted in accordance with the Declaration of Helsinki, and the project was approved by the Bioethics Committee of the Wroclaw Medical University (decision no.: KB-22/2021). Participation in the study was voluntary and anonymous. Participants were informed about the purpose of the study and provided informed consent. The questionnaire was made available in electronic form. The form was accessible for three weeks, using the Google Forms platform, with no time limit for completion, and the project coordinator was available for contact in case of doubts. The detailed research process is presented in Figure 1.

Anthropometric measurements included the assessment of body mass (using a Tanita body composition analyzer (Tanita BC-545N, Tokyo, Japan)) and height (using a stadiometer (Tanita HR-001, Tokyo, Japan), based on which the BMI index was determined.

### 2.2. KomPAN Questionnaire

Nutritional data were analyzed using the standardized KomPAN [19,20] questionnaire, consisting of four parts: (1) dietary habits; (2) food consumption frequency; (3) views on food and nutrition; and (4) lifestyle and personal data. According to the KomPAN procedure, the following indices were calculated:

Pro-Healthy Diet Index (pHDI-10)—accounting for 10 food groups with a potentially beneficial effect on health;

Non-Healthy Diet Index (nHDI-14)—accounting for 14 food groups with a potentially adverse effect on health.

These indices were calculated by summing the frequency of consumption (times/day) of the indicated 10 (pHDI-10) and 14 food groups (nHDI-14).

The Nutrition Knowledge Index (NKI) is estimated based on questions containing 25 statements about food and nutrition, with response options of “true”, “false”, or “unsure”, where 1 point is awarded for each correct answer, and 0 points for an incorrect answer or lack of knowledge. The total score reflects the participant’s level of nutritional knowledge as: insufficient (0–8 pts), sufficient (9–16 pts), or good (17–25 pts).

To determine the share of a healthy diet within the total diet of the subjects, the variable pHDI-10/nHDI-14 was introduced (omitting absolute index values), representing the quotient of the pHDI-10 and nHDI-14 values.

### 2.3. Pain and Injury Assessment

Injuries, pain complaints, sports supplementation, and the use of dietitian and physiotherapist services were analyzed using an original questionnaire. Areas of pain complaints were identified, and pain intensity was assessed (on a scale of 1–10). The frequency of injuries was estimated over the last 12 months and 7 days within specific body parts (neck and nape, shoulders, upper back, elbows, wrists and hands, lower back, hips and thighs, knees, ankles, and feet). An injury was defined as trauma to the musculoskeletal system causing pain or functional limitation. A variable describing the number of painful sites with a pain intensity of at least mild (≥3/10), moderate (≥5/10), or severe (≥7/10) was introduced for analysis. Total pain intensity denoted the sum of all pain intensities in sites indicated as painful. Average pain intensity was defined as the quotient of the total pain intensity and the number of sites where pain symptoms were indicated during the last 7 days. Due to the population-based nature of the study and the insufficient number of participants using specific categories of supplements (with the majority of the “users” group reporting supplementation primarily with vitamins and protein, and only a few individuals using other types of dietary supplements), it was not possible to conduct reliable analyses for individual supplement types. Therefore, supplementation was analyzed as a single aggregated variable. Group characteristics are presented in Table 1.

### 2.4. Statistical Analysis

Data were compiled using Microsoft Excel (version 16.77, Redmond, WA, USA) and analyzed using Statistica 13 (StatSoft, Kraków, Poland). The reliability analysis of the original questionnaire showed a Cronbach’s alpha coefficient in the range of 0.77–0.89. The Shapiro–Wilk test was used to assess the normality of data distribution (data did not follow a normal distribution). Descriptive statistics were calculated, including counts for categorical variables and medians with interquartile ranges (IQRs) for continuous variables. For comparisons involving two independent groups, the U Mann–Whitney test was used. When comparing a continuous variable across three or more independent groups, the Kruskal–Wallis test was applied. Due to the relatively small number of observations (*n* = 53) in relation to the number of potential predictors (risk of model overfitting), the multivariate models were not used. Statistical tests were conducted at a significance level of *p* ≤ 0.05. For each of the conducted tests, a power analysis was carried out to determine the statistical power.

## 3. Results

### 3.1. Dietary Supplement Use and Nutritional Habits

The comparison of nutritional habits (Table 2) between players using and not using dietary supplements shows that the former are characterized by a lower mean value of the n-HDI-14 index and a higher pHDI-10/n-HDI-14 ratio. This means that supplement users consume fewer unfavorable products (*p* = 0.01) and have a more favorable balance of pro-health to unhealthy products (*p* = 0.02). They also eat more meals per day (*p* < 0.001) and have fewer snacking episodes during the day (*p* < 0.001). No significant differences were found in the level of knowledge (*p* = 0.17) between the analyzed groups.

### 3.2. Dietary Supplement Use and Musculoskeletal Parameters

The comparison of musculoskeletal parameters (Table 3) shows that there are no significant differences in the number of injuries, either over 12 months (*p* = 0.87) or over 7 days (*p* = 0.58), between players using dietary supplements and those who did. Similarly, the groups did not differ in terms of total (*p* = 0.27) and average pain intensity (*p* = 0.67). There were also no differences among those reporting the number of painful sites with at least mild (*p* = 0.96) and severe intensity (*p* = 0.20). The only difference was present in the number of sites declared as having at least moderate pain intensity (*p* = 0.04), which means players using dietary supplements reported such complaints more frequently than non-users.

### 3.3. Nutritional Habits and Musculoskeletal Parameters

Spearman’s correlation analysis (Table 4) showed a statistically significant weak positive relationship between the number of injuries over 12 months and the pro-healthy diet index (*p* = 0.04, r = 0.28). Furthermore, the number of injuries over 7 days moderately positively correlated with the pHDI-10 value (*p* < 0.001, r = 0.53) and the pHDI-10/nHDI-14 ratio (*p* = 0.046, r = 0.40). A weak negative correlation was also observed between the declared number of sites with severe pain intensity and the number of meals consumed per day (*p* = 0.04, r = −0.35) and snacking frequency (*p* = 0.05, r = −0.34), as well as between severe pain intensity and snacking frequency (*p* = 0.03, r = −0.31). Additionally, the number of injuries over 7 days moderately positively correlated with NKI (*p* < 0.001, r = 0.45). A correlation was also observed between the number of sites with at least moderate pain intensity and NKI (*p* = 0.02, r = 0.32)

### 3.4. Use Professional’s Help in Comparison to Dietary and Musculoskeletal Factors

Table 5 presents dietary and injury variables that proved significantly associated with the use of dietitian or physiotherapist services. Regarding physiotherapist services, users showed a significantly higher intake index of pro-health products (pHDI-10; *p* = 0.02), a higher number of injuries in the last 7 days (*p* = 0.01), and higher total (*p* = 0.03) and average pain intensity (*p* < 0.001) compared to those not using physiotherapy. Differences in the consumption of unhealthy products (nHDI-14; *p* = 0.41) were not statistically significant, although a tendency towards higher values was observed in the group utilizing physiotherapist assistance (18.3 vs. 14.8).

Among players utilizing sports dietitian assistance, a significantly higher intake index of pro-health products (pHDI-10; *p* = 0.002), a higher number of injuries in the last 12 months (*p* = 0.02) and 7 days (*p* = 0.008), and a higher total pain intensity (*p* = 0.009) were found compared to those not utilizing dietary consultations (Table 5). Differences in the consumption of unhealthy products (nHDI-14; *p* = 0.39) and average pain intensity (*p* = 0.34) were not statistically significant.

## 4. Discussion

The aim of this study was to assess the relationship between supplementation, diet quality, nutritional awareness, use of dietitian and physiotherapist support, injury incidence and experienced musculoskeletal pain among AF players in Poland. The results allow for both the confirmation of some existing observations and the identification of several non-obvious, potentially new phenomena in this area. Given the relatively small sample size and the number of statistical comparisons conducted, the present analyses should be considered exploratory. Therefore, the findings should be interpreted with caution and regarded as hypothesis-generating rather than definitive.

**H1.**  *Supplement users are characterized by higher diet quality and greater nutritional awareness.*

The benefits of sports supplementation are well documented in cases of confirmed nutritional deficiencies, increased energy needs, the necessity for ergogenic action (e.g., caffeine, creatine), and—crucially for the discussed issue—support for post-exercise recovery [21,22]. The literature demonstrates that supplement users more often display a greater regularity in meal consumption [23] and greater care in product selection [24]. However, it should be noted that supplementation rarely functions as an isolated behavior. Our results are consistent with the concept of health behavior clustering, according to which people who take one action aimed at improving performance or recovery are also more likely to exhibit other beneficial eating habits. It can be assumed that the use of supplements in the study group is not only a response to deficiencies, but part of a broader strategy of diet control and optimization of athletic performance. More regular meals (*p* < 0.001) and less frequent snacking (*p* < 0.001) may indicate a higher level of nutritional awareness or greater commitment to the training process. This relationship has also been observed in other sports (team sports, strength sports [22] and elite sports [22,25]), suggesting that supplementation may be an indicator of a general focus on performance and health, rather than merely a tool to compensate for dietary deficiencies.

The obtained results also provide a more precise perspective, showing that within the group of supplement users, differences exist in specific nutritional behaviors. Specifically, AF players consume fewer unfavorable products (*p* = 0.01) and have a more favorable balance of pro-health to unhealthy products (*p* = 0.02). This does not mean that supplementation itself is unequivocally desirable. The positions of the IOC, AIS, and ISSN [22,26,27] emphasize that its use should be justified by actual need and based on scientific evidence. The AIS classification system indicates that many supplements have limited evidence of effectiveness, and decisions to take them are often motivated by marketing. In competitive sports, the risk of unintentional anti-doping rule violations, related, among other things, to contamination of preparations or incomplete control of their composition, should also be taken into account. Therefore, supplementation should be treated as an intervention requiring individual assessment and professional supervision, rather than as an indicator of a higher quality diet [28]. Regardless of this, our results may suggest that FA athletes who use supplements strive for a more conscious and comprehensive approach to nutrition. However, the lack of differences in nutritional knowledge that we observed does not confirm this. This may suggest that they strive for a more conscious and comprehensive approach to nutrition. However, the lack of differences in the level of nutritional knowledge observed in our study does not confirm this. In the literature, it is often assumed that supplementation co-occurs with higher nutritional awareness [29] and is the result of deepened theoretical knowledge [30]. Meanwhile, among the footballers we studied, supplementation does not always go hand in hand with this. Perhaps it is more of an element of a plan prepared by the coaching staff. It may reflect environmental recommendations or be a consequence of practical training experiences. For instance, it cannot be ruled out that it is used as a compensatory measure for previous health problems, high training loads, drops in form, or past injury—as noted by other researchers [31,32]. In such a case, supplementation may be a pragmatic support tool, i.e., an effect of the level of training commitment or the intensity of sports demands. This indicates that supplementation in the AF group is rather an indicator of a structured lifestyle, and not its main cause (it does not shape a consistent pattern of health behaviors but appears “incidentally”). This discovery enriches knowledge regarding the nutritional model typical for such an organized way of functioning [9].

**H2.**  *Supplement users differ from non-users in terms of the number of injured sites and the number of sites with a specific pain intensity.*

The results did not confirm the hypothesis regarding distinct differences in the number of injuries (either over 12 months or over 7 days) or total and average pain intensity between players using and not using supplementation. Such a lack of significant differences is consistent with findings from recent years [5,8,33], which demonstrate that supplementation plays only a supplementary role in injury prevention and should be considered within the framework of a broader planned nutritional and training intervention, rather than as a standalone injury prevention factor [34].

An interesting exception is the fact that AF players taking supplements reported painful areas with at least moderate pain intensity less frequently compared to non-users (*p* = 0.04), despite the lack of differences in the overall number of injuries and painful sites. This may indicate that supplementation primarily influences pain perception rather than actual injury incidence. According to findings by Miguel-Ortega [34], supplements such as omega-3, collagen, or vitamin D have anti-inflammatory and regenerative effects, alleviating the sensation of ailments, but do not change the mechanical risk of injury. This result suggests that supplementation may reduce pain symptoms accompanying training loads and indicates a direction requiring further research verification. It is worth noting that this phenomenon was not observed in players reporting severe pain complaints, which may result from the fact that such extensive ailments reflect significant overload, against which the action of anti-inflammatory and regenerative supplements is insufficient.

**H3.**  *A higher level of healthy nutritional behaviors (more regular meals, better diet, and less frequent snacking during the day) correlates with a lower number of injuries and lower pain intensity.*

The obtained results yielded several non-obvious observations. According to the current literature [34], a higher diet quality should be associated with lower injury rates and less frequent occurrence of pain. Indeed, a properly balanced diet (encompassing adequate supply of energy, protein, carbohydrates, and key micronutrients) plays a key role in maintaining tissue health, reducing inflammation, and supporting recovery processes [35]. However, in our study, weak positive correlations were found between the pro-healthy diet index (pHDI-10), the number of injuries (over 7 days and 12 months; *p* < 0.001 and *p* = 0.04, respectively) and between pHDI-10 and pHDI-10/nHDI-14—exclusively in the case of injuries sustained within 7 days (*p* < 0.001). This means that players who ate better reported more injuries. This finding is surprising and may have several potential explanations. However, the proposed explanations are speculative and require verification in studies that take into account individual training exposure indicators. First, an injury may serve as a “turning point” leading to the improvement of nutritional habits. Researchers [36] demonstrate that athletes, following an injury, more frequently declare readiness to introduce changes in the area of nutrition and recovery, treating these changes as an element supporting the return-to-fitness process. Secondly, those who care more about their diet may simultaneously train more intensely, which, according to the literature [37,38], increases exposure to overload stimuli and injury risk. Thirdly, individuals with higher nutritional awareness may more accurately identify and more frequently report pain symptoms and micro-injuries, which is also consistent with research [39].

Regardless of the interpretation, our results show that greater health self-awareness is associated with a higher reporting of somatic problems. The observed weak, negative correlations both between declarations of the number of sites with severe pain intensity and the number of meals consumed per day (*p* = 0.04) and snacking frequency (*p* = 0.03), as well as between the number of sites with at least moderate pain intensity and snacking frequency (*p* = 0.02), suggest that more frequent meal consumption and snacking may be associated with lower pain sensations. This may reflect the beneficial effect of a more even energy distribution during the day on the functioning of the musculoskeletal system, glycemic stability, and recovery processes.

**H4.**  *Utilizing the services of a physiotherapist after injury and a dietitian is associated with higher diet quality, lower pain intensity, and a higher number of injured sites.*

As expected, AF players utilizing the services of a dietitian and physiotherapist exhibited a higher diet quality (*p* = 0.002 and *p* = 0.02), which is fully consistent with the literature regarding the influence of professional support on improving nutritional habits [40,41]. However, similar to the correlations we presented earlier, observations regarding the link to injury rates are complex. Specifically, athletes utilizing physiotherapy and dietetics reported a higher number of injuries over the last 7 days (*p* = 0.008 and *p* = 0.01, respectively) and higher total pain intensity (*p* = 0.03 and *p* = 0.009). Those using exclusively the services of a physiotherapist declared a higher average pain intensity (*p* < 0.001), and those using exclusively dietary advice declared a higher number of injuries over 12 months (*p* = 0.02).

The possibility of a reverse direction of causality is of key interpretative importance here. It seems more likely that athletes experiencing injuries or severe pain are more likely to seek specialist support than that the use of such support is associated with increased injury rates. In addition, athletes with a higher training load—and therefore potentially more prone to overload—may also have more frequent access to physiotherapy and dietary care.

In this context, the observed relationships may reflect a reactive model of care rather than systematic, proactive prevention [42]. However, it cannot be determined whether limited access to preventive measures coexists with higher injury rates, or whether an increased number of injuries leads to more frequent use of support. Similarly, higher pain intensity among those using the services of a dietitian may be due to a selection effect—nutritional support may be sought more often by athletes who are more heavily trained [40].

In light of the cross-sectional nature of the study, professional support should therefore be interpreted with caution—not as a protective or risk factor, but as a potential indicator of higher training load or existing health problems. This constitutes a significant contribution to the literature, suggesting the need to implement these services not only reactively but also preventively [43].

## 5. Limitations

Despite the valuable insights provided by this study, several limitations must be acknowledged. First, the sample size was relatively small, and the unequal distribution across subgroups may have reduced the statistical power of the performed analyses. This imbalance limits the robustness of between-group comparisons and may increase the likelihood of both Type I and Type II errors. Given the exploratory nature of the study and the large number of statistical tests performed, the risk of false-positive findings cannot be excluded. Therefore, the statistically significant associations identified should be interpreted with a high degree of caution and should rather be treated as hypothesis-generating rather than definitive. We recommend, that future research should be made on larger and more balanced cohorts of AF players to increase the credibility and robustness of the research.

Another important limitation concerns the absence of data on potential confounding variables, such as player position, training load, playing experience, age, or previous injury history. These factors may substantially influence both injury prevalence and perceived musculoskeletal burden. Their omission limits the ability to control for alternative explanations and reduces internal validity. Future research should aim to incorporate these variables and apply more comprehensive analytical models to better isolate independent predictors.

Additionally, the study relied on a broad, functional definition of musculoskeletal injury based on participants’ self-reported pain and functional limitations. While this approach aligns with the study’s objective of assessing perceived musculoskeletal burden, it does not allow for clinical differentiation between acute injuries and overuse syndromes, nor does it enable the identification of specific etiologies. The lack of medical verification further precludes the confirmation of clinical diagnoses.

Additionally, dietary supplementation was analyzed as an aggregated variable. Given the heterogeneity of supplements and their varying physiological effects, this approach does not allow for the identification of specific substances that may influence injury load, but it treats supplementation use as a general health-related behavior. Future studies should focus on categorizing supplements by type, dosage and frequency of taking to provide more detailed conclusions.

The retrospective and self-reported nature of the data introduces the potential for recall bias, particularly with respect to the 12-month reporting period. Although the inclusion of a 7-day recall period helped to capture a more current health status and partially mitigate this limitation, inaccuracies in long-term recall cannot be ruled out. Finally, the cross-sectional design prevents any causal inferences regarding the relationships observed, as temporal directionality cannot be established.

Future studies should:

- Recruit larger and more evenly distributed samples to enhance statistical power and subgroup comparability;
- Implement longitudinal study designs to allow for causal inference and temporal analysis of injury development;
- Include key confounding variables such as age, training load, player position, and playing experience to improve internal validity;
- Include the variables of supplement type, dosage and frequency of taking to provide more detailed data;
- Utilize objective measurement tools (e.g., clinical assessments, medical records, workload monitoring systems) alongside self-reported data to strengthen diagnostic accuracy;
- Differentiate between acute injuries and overuse syndromes to enable more precise epidemiological and clinical interpretations;
- Replicate findings in diverse populations and competitive levels to improve generalizability.

Addressing these methodological considerations will contribute to a more comprehensive understanding of musculoskeletal burden and its determinants, ultimately supporting the development of targeted prevention and intervention strategies.

## 6. Conclusions

The analysis of the results suggests that both supplementation and the use of dietitian and physiotherapist services do not result from preventive actions, but rather reflect increased training loads and greater susceptibility to overload among AF players. While it is true that those using supplementation present better nutritional habits, these differences do not seem to stem from a higher level of nutritional knowledge, but from a more structured lifestyle and practical training experiences. The relationship between a “healthier” diet and a higher number of injuries suggests that it is a greater exposure to loads, not the mode of nutrition, that lies behind increased injury rates. Associations regarding pain perception and the number of injuries among supplementing individuals additionally support this interpretation. Although supplement use was associated with lower levels of perceived pain, no significant association was observed with the reported number of injuries. This finding may suggest that supplementation co-occurs more closely with subjective perceptions of discomfort than with the objective frequency of injuries.

Given the cross-sectional nature of the study, the results obtained do not allow for causal conclusions or clear recommendations for intervention. However, they may serve as an important signal for practitioners working with athletes. The observed co-occurrence of professional support with higher pain intensity and a greater number of injuries suggests the need for more careful monitoring of training load and the timing of specialist care. The results may indicate the superiority of the reactive model over the preventive model, which is worth verifying in longitudinal studies. From a practical perspective, it seems reasonable to strengthen systematic health education, individualize support, and coordinate activities between the coach, physical therapist, and dietitian. However, these recommendations are cautious and require confirmation in studies with a more advanced methodological design.

## Figures and Tables

**Table 1:** Characteristics of the study.

Variable		Mean (Median) [IQR] *N* = 53
Age		22.1 (21.0) [19.0–24.0]
Pain intensity		5.01 (5.0) [4.0–6.0]
**KomPAN**		
pHDI-10		23.2 (22.5) [18.8–27.4]
nHDI-14		17.4 (17.5) [10.3–25.6]
pHDI-10/nHDI-14		1.7 (1.3) [0.8–2.2]
NKI		11.0 (10.0) [10.0–12.0]
Number of meals per day		4.2 (4.0) [4.0–5.0]
Number of snacking episodes per day		0.8 (0.5) [0.14–1.0]
**Data regarding pain symptoms**		
Number of injuries in 12 months		3.7 (3.0) [2.0–5.0]
Number of injuries in 7 days		2.2 (2.0) [1.0–3.0]
Total pain intensity		18.1 (15.0) [10.0–25.0]
Average pain intensity		5.0 (5.0) [4.0–6.0]
Number of painful sites with intensity	≥3	3.6 (3.0) [2.0–5.0]
	≥5	1.9 (2.0) [1.0–3.0]
	≥7	0.4 (0.0) [0.0–0.0]

Note: pHDI-10—pro-Healthy Diet Index; nHDI-14—non-Healthy Diet Index; NKI—Nutrition Knowledge Index.

**Table 2:** Indicators of diet quality and nutritional awareness depending on the use or non-use of supplements by American football players.

	Dietary Supplement Use								
	Yes, *N* = 37			No, *N* = 16			Yes vs. No		
	Mean	Median	IQR	Mean	Median	IQR	*p*-Value	|Z|	1−β
pHDI-10	23.6	19.4	12.2–36.4	22.8	22.7	21.7–26.4	0.88	0.15	0.06
nHDI-14	13.7	13.4	8.1–17.5	20.8	19.4	12.6–25.9	**	2.5	0.88
pHDI-10/nHDI-14	2.1	1.8	1.0–2.9	1.3	1.0	0.8–1.7	*	2.3	0.79
NKI	11.8	11.0	10.0–15.0	10.3	10.0	10.0–11.0	0.17	1.4	0.83
Number of meals per day	4.4	4.0	4.0–5.0	3.9	4.0	4.0–4.0	***	3.4	0.87
Snacking episodes per day	0.6	0.5	0.14–0.5	1.0	1.0	0.32–2.0	***	3.4	0.50

Note: pHDI-10—pro-Healthy Diet Index; nHDI-14—non-Healthy Diet Index; NKI—Nutrition Knowledge Index; * p≤0.05; ** p≤0.01; *** p≤0.001.

**Table 3:** Number of injuries, painful sites, and pain intensity among American football players using and not using supplementation.

		Dietary Supplement Use								
		Yes, *N* = 37			No, *N* = 16			Yes vs. No		
		Mean	Median	IQR	Mean	Median	IQR	*p*-Value	|Z|	1−β
Number of injuries in last:	12 months	3.7	3.0	3.0–5.0	3.6	3.0	2.0–5.5	0.87	0.2	0.44
	7 days	2.1	2.0	1.0–3.0	2.3	2.0	1.5–3.0	0.58	0.5	0.07
Number of painful sites (intensity):	≥3	3.5	3.0	3.0–5.0	3.6	3.0	2.0–5.5	0.96	0.1	0.05
	≥5	1.7	1.0	0.0–3.0	2.0	2.0	2.0–3.0	*	2.1	0.10
	≥7	0.4	0.0	0.0–0.0	0.0	0.0	0.0–1.0	0.20	1.3	0.37
Total pain intensity		17.8	15.0	9.0–25.0	14.5	10.0	10.0–29.5	0.27	1.1	0.22
Average pain intensity		5.0	5.0	3.3–6.3	5.0	4.9	4.9–5.2	0.67	0.4	0.05

Note: * p≤0.05.

**Table 4:** Spearman correlation between diet quality indices, knowledge, and nutritional behaviors (frequency of meals, snacking episodes during the day) and the number of injuries and pain intensity.

		pHDI-10		nHDI-14		pHDI-10/nHDI-14		NKI		Number of Meals Per Day		Number of Snacking Episodes Per Day	
		*p*	r	*p*	r	*p*	r	*p*	r	*p*	r	*p*	r
Number of injuries in last 12 months		*	0.28	0.52	0.09	0.47	0.10	0.12	0.21	0.35	0.13	0.54	−0.08
Number of injuries in last 7 days		***	0.53	0.96	0.006	***	0.46	***	0.45	0.94	0.009	0.33	0.14
Number of painful sites with intensity	≥3	0.10	0.23	0.44	0.11	0.69	0.06	0.14	0.20	0.41	0.11	0.34	−0.13
	≥5	0.16	0.19	0.44	0.11	0.37	0.13	*	0.32	0.07	−0.25	0.97	−0.005
	≥7	0.70	0.05	0.45	0.11	0.89	0.02	0.63	−0.07	*	−0.28	*	−0.30
Total pain intensity		*	0.30	0.34	0.13	0.19	0.18	*	0.34	0.67	−0.06	0.48	−0.10
Average pain intensity		0.20	0.18	0.47	−0.10	**	0.36	0.11	0.22	0.18	−0.19	*	−0.31

Note: pHDI-10—pro-Healthy Diet Index; nHDI-14—non-Healthy Diet Index; NKI—Nutrition Knowledge Index; * p≤0.05; ** p≤0.01; *** p≤0.001.

**Table 5:** Selected dietary indicators and pain intensity among players using or not using the assistance of a physiotherapist after injury and a sports dietitian.

Factors	Use of Physiotherapist Services After the Injury								
	Yes, *N* = 40			No, *N* = 13			Yes vs. No		
	Mean	Median	IQR	Mean	Median	IQR	*p*-Value	|Z|	1−β
pHDI-10	24.7	23.3	19.4–27.8	18.7	20.6	12.2–20.8	*	2.4	0.63
n-HDI-14	18.3	16.8	9.6–25.9	14.8	17.5	11.3–17.5	0.41	0.8	0.37
Number of injuries in last 12 months	3.68	4.0	2.0–5.5	3.6	3.0	3.0–3.0	0.92	0.1	0.05
Number of injuries in last 7 days	2.5	2.0	1.0–3.0	1.3	1.0	0.0–1.0	**	2.5	0.63
Total pain intensity	19.6	19.5	10.0–25.0	13.5	9.0	9.0–10.0	*	2.2	0.47
Average pain intensity	5.5	5.0	4.9–6.1	3.5	3.0	3.0–3.3	***	4.3	0.99
	**Use of dietitian services after the injury**								
	**Yes, *N* = 16**			**No, *N* = 37**			**Yes vs. No**		
pHDI-10	28.7	25.6	23.3–36.4	20.8	20.8	12.2–26.4	**	3.1	0.89
n-HDI-14	15.3	15.4	8.1–19.4	18.3	17.5	10.6–25.9	0.39	0.9	0.22
Number of injuries in last 12 months	4.6	5.0	3.5–6.0	3.3	3.0	2.0–4.0	*	2.4	0.60
Number of injuries in last 7 days	3.2	3.0	2.0–4.5	1.7	2.0	1.0–3.0	**	2.7	0.82
Total pain intensity	23.3	23.0	15.5–27.5	15.8	10.0	9.0–25.0	**	2.6	0.58
Average pain intensity	5.5	5.0	3.8–6.4	4.8	5.0	4.8–5.3	0.34	0.9	0.27

Note: pHDI-10—pro-Healthy Diet Index; nHDI-14—non-Healthy Diet Index; * p≤0.05; ** p≤0.01; *** p≤0.001.

## Data Availability

The datasets used and analyzed during the current study are available from the corresponding author upon reasonable request. The data are not publicly available due to the inclusion of information that could compromise the privacy of the research participants in accordance with the decision of the Ethics Committee of the Wroclaw Medical University.

## Abbreviations

The following abbreviations are used in this manuscript:

AF	American Football
pHDI-10	Pro-Healthy Diet Index
nHDI-14	Non-Healthy Diet Index
NKI	Nutrition Knowledge Index
IQR	Interquartile Range
